# American Foulbrood in the Czech Republic: ERIC II Genotype of *Paenibacillus Larvae* Is Prevalent

**DOI:** 10.3389/fvets.2021.698976

**Published:** 2021-08-18

**Authors:** Jana Biová, Jaroslav Bzdil, Silvie Dostálková, Marek Petřivalský, Jan Brus, Elena Carra, Jiří Danihlík

**Affiliations:** ^1^Department of Biochemistry, Faculty of Science, Palacký University Olomouc, Olomouc, Czechia; ^2^State Veterinary Institute Olomouc, Olomouc, Czechia; ^3^Department of Geoinformatics, Faculty of Science, Palacký University Olomouc, Olomouc, Czechia; ^4^Experimental Zooprophylactic Institute in Lombardy and Emilia Romagna (IZSLER), Brescia, Italy

**Keywords:** ERIC genotype, Epizootology, honey bee *(Apis mellifera L.)*, Europe, Dangerous infectious disease

## Abstract

American foulbrood (AFB) is a dangerous disease of honeybees (*Apis mellifera*) caused by the spore-forming bacterium *Paenibacillus larvae*. According to the ERIC (enterobacterial repetitive intergenic consensus) classification, five genotypes are distinguished, i.e., I, II, III, IV, and V, which differ in their virulence and prevalence in colonies. In the Czech Republic, AFB prevalence is monitored by the State Veterinary Administration; however, the occurrence of specific *P. larvae* genotypes within the country remains unknown. In this study, our aim was to genotype field *P. larvae* strains collected in the Czech Republic according to the ERIC classification. In total, 102 field isolates from colonies with AFB clinical symptoms were collected from various locations in the Czech Republic, and the PCR genotypization was performed using ERIC primers. We confirmed the presence of both ERIC I and II genotypes, while ERIC III, IV, and V were not detected. The majority of samples (*n* = 82, 80.4%) were identified as ERIC II, while the ERIC I genotype was confirmed only in 20 samples (19.6%). In contrast to other European countries, the ERIC II genotype is predominant in Czech honeybee colonies. The ERIC I genotype was mostly detected in border regions close to Poland, Slovakia, and Austria.

## Introduction

American foulbrood (AFB) is defined as a dangerous contagious bacterial disease of honeybee (*Apis mellifera*) brood (1, 2). AFB is caused by the spore-forming bacterium *Paenibacillus larvae* (1, 3). The disease has great direct and indirect economic and ecological impacts because it has limited possibilities of treatment, e.g., prohibited use of antibiotics in the EU (4, 5). The disease affects the bee larvae during the early developmental stages, whereas adult bees only transmit the infectious spores of *P. larvae*. When bee larvae consume food sources contaminated by *P. larvae* endospores, these spores germinate in the larval intestine. The youngest larvae (12–36 h after egg hatching) represent the most sensitive stage to the spores (1) when even a very low dose of spores (median lethal dose, LD_50_ ~9 bacteria per larvae) is lethal in “*in vitro*” conditions (6). The dead larvae degrade into typical brown, sticky, and partly fluid “jelly,” which is the primary clinical symptom for AFB diagnosis (4).

Microbiological and biochemical differences between genotypes of enterobacterial repetitive intergenic consensus (ERIC) *P. larvae* strains have been previously described among phenotypically and morphologically different *P. larvae* cultures (7, 8) and can result in diverse sensitivity of spores to high temperatures (9) or production of bacterial toxins (10, 11).

Importantly, the infection progression differs between ERIC I and II genotypes. Larvae infected with ERIC I genotype died 12 days after exposure, whereas infection with the ERIC II genotype caused larval deaths after 7 days (7, 8). Furthermore, faster colonization of the digestive tract of larvae by the ERIC II genotype compared to the ERIC I genotype was observed (12). The prevalence of all ERIC genotypes varies among countries and continents. Genersch et al. (8) performed genotypization of *P. larvae* strains collected in Germany, Finland, and Sweden; however, only ERIC I and II genotypes were detected in these European countries. Later studies in Austria and Italy also confirmed the presence of only ERIC I and II genotypes (13, 14). In a survey of South and North American countries, only ERIC I genotypes were found among Argentinian and Uruguayan strains (15). A previous analysis by Alippi et al. (16) explored four different clusters of ERIC I genotypes in Buenos Aires province in Argentina, i.e., BOX A, B, C, and D. Samples from the USA were confirmed only as ERIC I genotypes (17). The ERIC V genotype was newly detected in a honey sample in Spain (7).

Similar to many other organisms, the *P. larvae* genome contains preserved repetitive DNA sequences, which vary in number and length within a species (18). Specific primer sets and PCR conditions were designed to identify repetitive sequences and were tested for *P. larvae* genotypization, such as the BOX system represented by BOX A1R primers (18), the REP system with MBO REP1 primers by Versalovic et al. (18), and the ERIC system exploiting ERIC1R and ERIC2 primers (18). The ERIC system was originally established to detect conserved repetitive sequences in enterobacteria (ERIC = enterobacterial repetitive intergenic consensus), but later, it has been shown to be useful also in *P. larvae* genotypization, as it can distinguish five genotypes of *P. larvae* (ERIC I–ERIC V) (7, 19).

The State Veterinary Administration systematically monitors AFB in the Czech Republic. There were 242, 152, and 113 AFB outbreaks recorded in 2016, 2017, and 2018, respectively (20). Here, we aimed to genotype the field isolates of *P. larvae* from affected apiaries and to provide the first report on the occurrence and distribution of individual ERIC genotypes of *P. larvae* in the Czech Republic.

## Materials and Methods

### Bacterial Isolates

**Figure 1:**
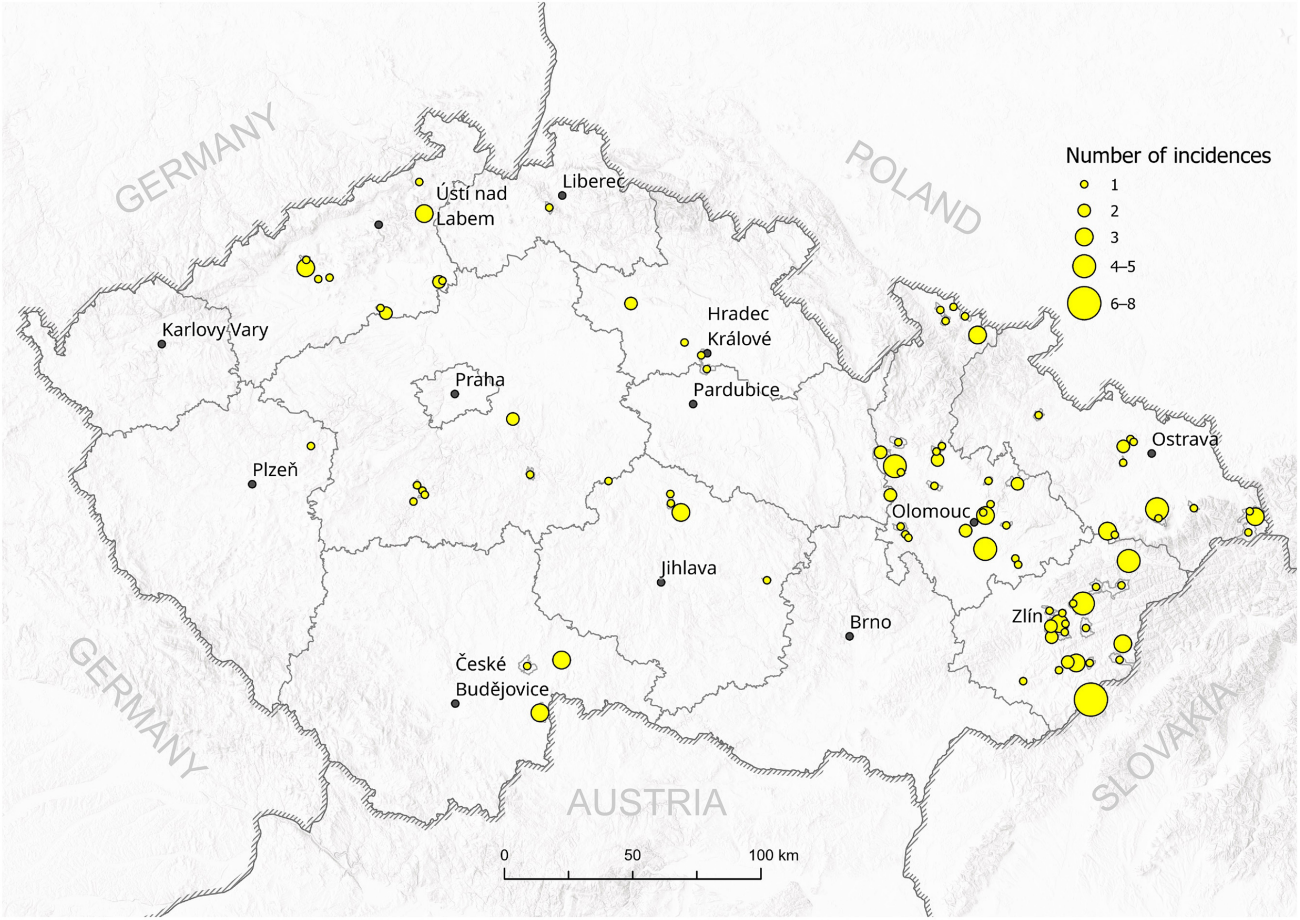
Total incidences of AFB outbreaks in the Czech Republic between 2016 and 2017. The number of outbreaks in particular districts is represented by the size of the circle.

A total of 102 *P. larvae* field isolates were collected and provided by the regional State Veterinary Institutes in Olomouc, Jihlava, and Prague (Czech Republic) for genotyping. The isolates were collected from honeybee colonies with AFB clinical symptoms between October 2016 and May 2017 as part of routine inspections. Figure 1 shows the total number of AFB incidences in districts in the Czech Republic (data kindly provided by the State Veterinary Administration, Czech Republic). Reference strains of *P. larvae* ERIC I (CCUG 48979) and ERIC II (CCUG 48972) genotypes were obtained from Eva Forsgren from SLU (Uppsala, Sweden). The positive controls for ERIC III–V were not available for us.

### Cultivation of *P. larvae*

The hive debris samples (*n* = 84) or suspected brood samples (*n* = 18) were collected from infected apiaries. The hive debris consists of wax chewed by bees and fallen down on hive bottom. The hive debris samples were processed and cultured by the standard Tween 80 method (21). The brood samples were cultured using a bacteriological loop dipped into the suspected cells and microbiologically cultured as follows: the inoculum of both types of materials was in parallel cultivated on one plate with meat peptone blood agar (MPBA) with 5% of sheep blood (Trios, Prague, Czechia) and two plates of MYPPn agar with glucose (Trios, Prague, Czechia) at 37°C for 5–8 days. Confirmation of pure *P. larvae* isolates was performed by MALDI-TOF MS biotyper with α-cyano-4-hydroxycinnamic acid as a matrix (Bruker Daltonics GmBH, Bremen, Germany). Validation of bacteriological media and MALDI-TOF MS method was performed using *P. larvae* reference strain CAPM 5875 (Veterinary Research Institute, Brno, Czech Republic).

### Genotyping of *P. larvae*

The procedure for DNA isolation was performed as described in Bassi et al. (13) with small modifications. Bacterial colonies from the MYPPn medium were suspended in 1 ml of water and afterward centrifuged (1 min at 12,000 × g), 800 μl of supernatant was discarded. The remaining supernatant and pellet were mixed with 200 μl InstaGene^TM^ matrix (Bio-Rad, Hercules, CA, USA) and incubated for 30 min at 56°C and mixed at 1,400 rpm. Then the mixture was vortexed for 10 s and reincubated for 8 min at 100°C, with mixing at 1,400 rpm, then again centrifuged (3 min at 12,000 × *g*). Part of the supernatant was removed to a new microtube and diluted to the concentration of 20 ng/μl DNA. The DNA was quantified by a Synergy HT microplate reader (BioTek, Bad Friedrichshall, Germany).

The endpoint PCR was performed with FastStart Taq DNA polymerase dNTPack (Roche, Basel, Switzerland) as follows (volumes given per one reaction): 2.5 μl 10 × PCR buffer without MgCl_2_, 2.5 μl 25 mM MgCl_2_, 0.63 μl 10 mM dNTPs, 2.5 μl 10 μM ERIC1R and ERIC2 primers [for the primer sequences, see Genersch et al. (8)], 8.87 μl PCR water, 0.5 μl FastStart Taq DNA Polymerase, and 5 μl of extracted DNA. DNA extracted from reference ERIC I and II strains were used as positive controls for the respective genotypes, whereas water was used as the negative control. The PCR cycle was set up as follows: 5 min 95°C (60 s 95°C, 60 s 53°C, 3 min 72°C) × 50, 5 min 72°C, ∞ 4°C. The PCR products were visualized by gel electrophoresis (80 V, 1 h) in 0.8% agarose gel in 1 × TAE buffer, with ethidium bromide (100 μl 0.07% to 100 ml of the gel).

Typical PCR fragments shared by both ERIC I and ERIC II strains are approximately 970 bp long, while ERIC I lacks the PCR fragment of about 2,800 bp observed in the analysis of the ERIC II genotype. The ERIC III genotype is characterized by two PCR products migrating between 1,500 and 2,000 bp and one strong band around 600 bp. In contrast, the ERIC IV genotype provides a typical pattern with the largest DNA fragment with an approximate size between 1,000 and 1,200 bp and no fragments above this size. Two PCR products migrating between 1,500 and 2,000 bp and an additional 1,200-bp DNA fragment are typical for the ERIC V genotype (7, 8).

## Results

### Genotyping of *P. larvae* Isolates

**Figure 2:**
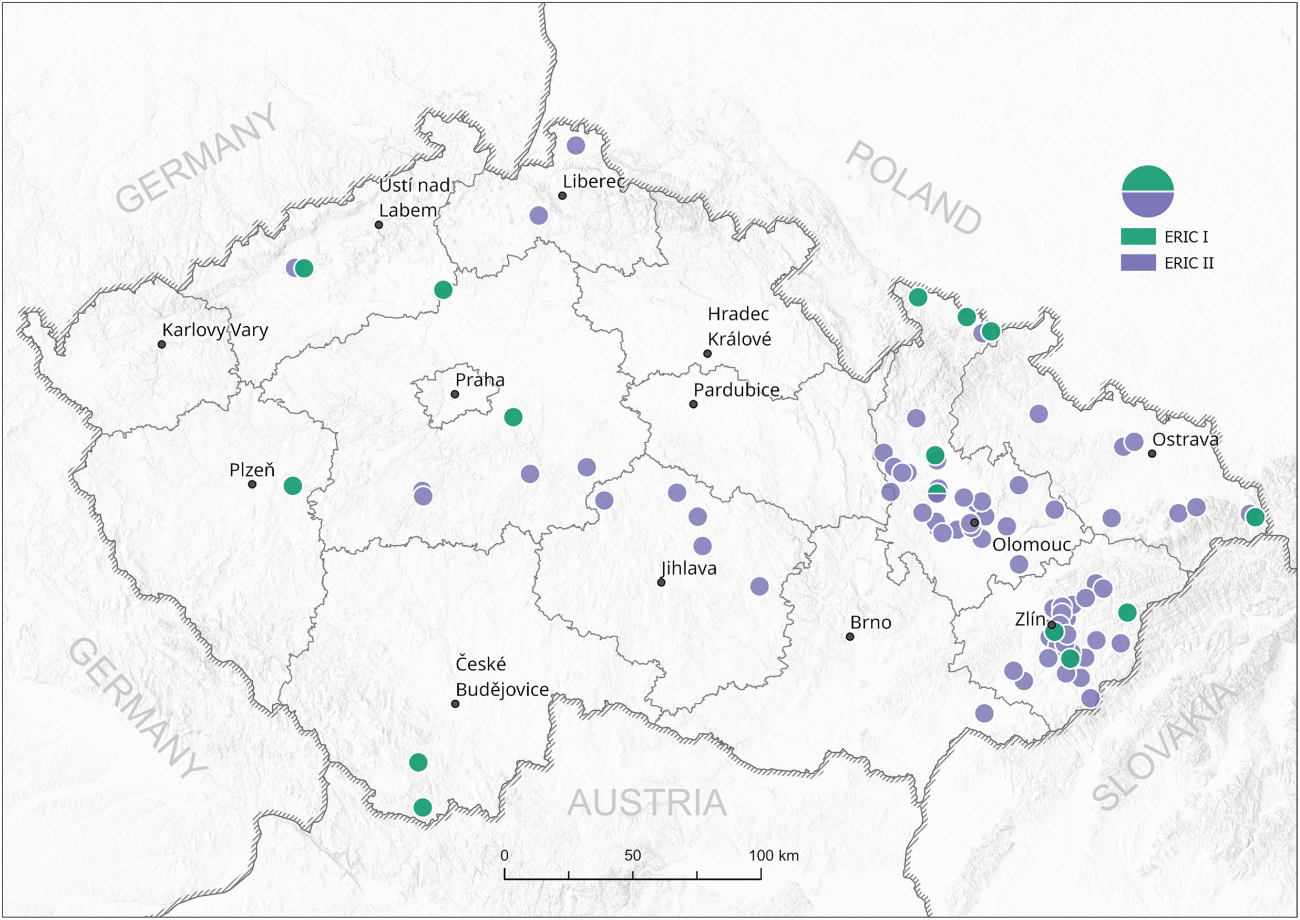
Spread of *P. larvae* genotypes ERIC I and II in the Czech Republic. Green circles—ERIC I, violet circles—ERIC II; circles represent one or more field isolates.

All 102 isolates were PCR genotyped by using specific ERIC primers. Only 20 isolates (19.6%) were classified as the ERIC I genotype, in contrast to 82 isolates (80.4%) identified as the ERIC II genotype. Typical patterns for ERIC III, IV, and V were not detected; thus, we assume that these were not present among analyzed samples at the time of conducting the study (Supplementary Material S1). Field isolates were collected from different regions of the Czech Republic and covered almost all regions with active outbreaks of AFB. There is an evident higher density of AFB outbreaks in the eastern part of the country, where AFB has been a long-term issue for the local beekeepers in previous years (Figure 1). On the other hand, AFB outbreaks in the central and western parts of the Czech Republic are relatively rare. The ERIC I genotype was mostly detected in two different areas in the southern and northern parts of the country. Interestingly, ERIC II was confirmed mostly in the central districts (Figure 2).

## Discussion

AFB has historically occurred in the Czech Republic with a higher prevalence in the eastern part of the country, where it has caused significant losses for local beekeepers (20). We sampled the field isolates at the turn of the years 2016 and 2017. The majority of field isolates were collected in 2017 (*n* = 98) when the State Veterinary Administration recorded in total 152 AFB outbreaks (20). Thus, our analysis provides a 64.4% coverage of all detected AFB outbreaks in the Czech Republic in 2017.

So far, detailed information about the occurrence of *P. larvae* strains in Czech Republic has been missing; thus, we aimed to perform the genotypization of *P. larvae* strains with the ERIC primers generally used for *P. larvae* classification (8). In agreement with other European studies on *P. larvae* ERIC genotypization, only the ERIC I and ERIC II genotypes were found among Czech field isolates. Surprisingly, the ERIC II genotype was detected in 80% of the samples, whereas the ERIC I genotype mostly occurred in samples collected close to the Czech national borders with Austria, Poland, and Slovakia. For comparison, 58% of Austrian *P. larvae* isolates belonged to the ERIC I and 42% of isolates to the ERIC II genotype. On the other hand, similar to our results on Czech isolates, ERIC III and ERIC IV genotypes have not been confirmed among Austrian strains (14). Detailed data from Poland and Slovakia are not currently available; however, Morrissey et al. (22) mentioned that ERIC II has not been found in Poland. The ERIC I genotype also prevails in other countries (22), e.g., Italy (13).

Genersch et al. (8) first reported slower disease progression if larvae are infected with ERIC I compared to ERIC II, and these findings were also confirmed by Beims et al. (7). We can hypothesize that this finding could be connected with reports of several veterinary inspectors, who reported difficulties with finding AFB clinical symptoms on larvae, because of spotty brood and missing dead or infected larvae under capes. Thus, the ERIC II major prevalence and faster death of larvae in combination with the hygienic behavior of bees (23) can result in problematic recognition of clinical symptoms and, thus, cause delayed confirmation of AFB disease at the clinical level. This research brings new insight on the situation in the Czech Republic and could help bee inspectors to find a new approach in searching for clinical symptoms in infected colonies with more virulent ERIC II genotype, e.g., uncapped or capped brood in early stages.

## Data Availability Statement

The original contributions presented in the study are included in the article/Supplementary Material, further inquiries can be directed to the corresponding author/s.

## Author Contributions

JBi performed the genotypization and writing manuscript. JBz collected the samples. SD and MP performed critical revision of manuscript. JBr geoinformatical analysis. EC method optimization. JD final approval of the version to be published and conception or design of the work. All authors contributed to the article and approved the submitted version.

## Conflict of Interest

The authors declare that the research was conducted in the absence of any commercial or financial relationships that could be construed as a potential conflict of interest.

## Publisher's Note

All claims expressed in this article are solely those of the authors and do not necessarily represent those of their affiliated organizations, or those of the publisher, the editors and the reviewers. Any product that may be evaluated in this article, or claim that may be made by its manufacturer, is not guaranteed or endorsed by the publisher.
