# Corrigendum: American Foulbrood in the Czech Republic: ERIC II Genotype of *Paenibacillus Larvae* Is Prevalent

**DOI:** 10.3389/fvets.2021.807222

**Published:** 2021-12-06

**Authors:** Jana Biová, Jaroslav Bzdil, Silvie Dostálková, Marek Petřivalský, Jan Brus, Elena Carra, Jiří Danihlík

**Affiliations:** ^1^Department of Biochemistry, Faculty of Science, Palacký University Olomouc, Olomouc, Czechia; ^2^State Veterinary Institute Olomouc, Olomouc, Czechia; ^3^Department of Geoinformatics, Faculty of Science, Palacký University Olomouc, Olomouc, Czechia; ^4^Experimental Zooprophylactic Institute in Lombardy and Emilia Romagna (IZSLER), Brescia, Italy

**Keywords:** ERIC genotype, Epizootology, honey bee *(Apis mellifera L.)*, Europe, Dangerous infectious disease

In the original article, there was an error in the affiliation of Jiří Danihlík. Instead of Affiliation 2, he should be affiliated with Affiliation 1: “Department of Biochemistry, Faculty of Science, Palacký University Olomouc, Olomouc, Czechia.”

The authors apologize for this error and state that this does not change the scientific conclusions of the article in any way. The original article has been updated.

## Publisher's Note

All claims expressed in this article are solely those of the authors and do not necessarily represent those of their affiliated organizations, or those of the publisher, the editors and the reviewers. Any product that may be evaluated in this article, or claim that may be made by its manufacturer, is not guaranteed or endorsed by the publisher.

