# Supplementary material for: American Foulbrood in the Czech Republic: ERIC II Genotype of Paenibacillus Larvae Is Prevalent
Source: Front Vet Sci. 2021 Aug 18;8:698976. doi: 10.3389/fvets.2021.698976 (PMC8416417; doi:10.3389/fvets.2021.698976)
Supplement: Supplementary file 1 [file Data_Sheet_1.PDF]

Supplementary Table S1: Sample IDs, locations and recorded *P. larvae* genotype

| Sample ID | Date of collection | Sample type | Village | District | ERIC genotype |
|---|---|---|---|---|---|
| 1 | 14.02.2017 | Debris | Nížkov | Žďár nad Sázavou | II |
| 2 | 18.01.2017 | Debris | Osečná | Liberec | II |
| 3 | 10.03.2017 | Debris | Rokycany | Rokycany | I |
| 4 | 14.02.2017 | Debris | Županovice | Příbram | II |
| 5 | 14.02.2017 | Debris | Hubenov u Borotic | Příbram | II |
| 6 | 08.03.2017 | Debris | Vyžlovka | Kolín | I |
| 7 | 14.02.2017 | Debris | Vyšší Brod | Český Krumlov | I |
| 8 | 03.03.2017 | Debris | Most | Most | I |
| 9 | 14.02.2017 | Debris | Chotěboř | Havlíčkův Brod | II |
| 10 | 14.02.2017 | Debris | Vyšší Brod | Český Krumlov | I |
| 11 | 14.02.2017 | Debris | Vyšší Brod | Český Krumlov | I |
| 12 | 14.02.2017 | Debris | Vyšší Brod | Český Krumlov | I |
| 13 | 14.02.2017 | Debris | Ledeč nad Sázavou | Havlíčkův Brod | II |
| 14 | 06.04.2017 | Debris | Štětí | Litovel | I |
| 15 | 04.04.2017 | Debris | Divišov u Benešova | Benešov | II |
| 16 | 15.02.2017 | Debris | Javorník | Jeseník | I |
| 17 | 15.02.2017 | Debris | Lubno | Frýdek-Místek | II |
| 18 | 15.02.2017 | Debris | Lubno | Frýdek-Místek | II |
| 19 | 15.02.2017 | Debris | Lubno | Frýdek-Místek | II |
| 20 | 15.02.2017 | Debris | Lubno | Frýdek-Místek | II |
| 21 | 15.02.2017 | Debris | Zděchov | Vsetín | I |
| 22 | 14.02.2017 | Debris | Větřní | Český Krumlov | I |
| 23 | 14.02.2017 | Debris | Větřní | Český Krumlov | I |
| 24 | 14.02.2017 | Debris | Větřní | Český Krumlov | I |
| 25 | 14.02.2017 | Debris | Vranová Lhota | Svitavy | II |
| 26 | 14.02.2017 | Debris | Vranová Lhota | Svitavy | II |
| 27 | 14.02.2017 | Debris | Vranová Lhota | Svitavy | II |
| 28 | 14.02.2017 | Debris | Havlíčkova Borová | Havlíčkův Brod | II |
| 29 | 14.02.2017 | Debris | Havlíčkova Borová | Havlíčkův Brod | II |
| 30 | 15.02.2017 | Debris | Litovel | Olomouc | I |
| 31 | 14.02.2017 | Debris | Křižanov | Žďár nad Sázavou | II |
| 32 | 11.05.2017 | Brood sample | Ratiboř | Vsetín | II |
| 33 | 11.05.2017 | Brood sample | Raškovice | Frýdek-Místek | II |
| 34 | 11.05.2017 | Brood sample | Písečná | Frýdek-Místek | I |
| 35 | 11.05.2017 | Brood sample | Ondřejovice | Jeseník | II |
| 36 | 11.05.2017 | Brood sample | Droždín | Olomouc | II |
| 37 | 17.05.2017 | Brood sample | Kozlovice | Nový Jičín | II |
| 38 | 18.05.2017 | Brood sample | Troubelice | Olomouc | II |
| 39 | 18.05.2017 | Brood sample | Nová Hradečná | Šumperk | I |
| 40 | 14.10.2016 | Debris | Želatovice | Přerov | II |
| 41 | 18.01.2017 | Brood sample | Nové Sady | Olomouc | II |
| 42 | 05.02.2017 | Debris | Nevšová | Zlín | II |
| 43 | 13.02.2017 | Debris | Veškv-Strážnice | Hodonín | II |

| | | | | | |
|---|---|---|---|---|---|
| 44 | 13.02.2017 | Debris | Veselí | Šumperk | II |
| 45 | 15.02.2017 | Debris | Rudice | Uherské Hradiště | II |
| 46 | 15.02.2017 | Debris | Štěpánov | Olomouc | II |
| 47 | 15.02.2017 | Debris | Vilémov | Olomouc | II |
| 48 | 15.02.2017 | Debris | Drahanovice | Olomouc | II |
| 49 | 15.02.2017 | Debris | Grygov | Olomouc | II |
| 50 | 15.02.2017 | Debris | Hněvotín | Olomouc | II |
| 51 | 15.02.2017 | Debris | Lašťany | Olomouc | II |
| 52 | 15.02.2017 | Debris | Náměšť na Hané | Olomouc | II |
| 53 | 15.02.2017 | Debris | Olomouc | Olomouc | II |
| 54 | 15.02.2017 | Debris | Slatinice | Olomouc | II |
| 55 | 14.10.2016 | Debris | Mohelnice | Šumperk | II |
| 56 | 05.12.2016 | Debris | Velké Kunětice | Jeseník | I |
| 57 | 15.02.2017 | Debris | Březůvky | Zlín | II |
| 58 | 15.02.2017 | Debris | Provodov | Zlín | II |
| 59 | 21.02.2017 | Debris | Litovel | Olomouc | II |
| 60 | 21.03.2017 | Debris | Bojkovice | Uherské Hradiště | II |
| 61 | 21.02.2017 | Debris | Olomouc | Olomouc | II |
| 62 | 27.02.2017 | Debris | Návsí | Frýdek-Místek | II |
| 63 | 21.02.2017 | Debris | Bohuňovice | Olomouc | II |
| 64 | 21.02.2017 | Debris | Horní Životice | Bruntál | II |
| 65 | 21.02.2017 | Debris | Daskabát | Olomouc | II |
| 66 | 08.03.2017 | Debris | Hvozdná | Zlín | II |
| 67 | 08.03.2017 | Debris | Zlín - Příluk | Zlín | II |
| 68 | 08.03.2017 | Debris | Březnice | Zlín | II |
| 69 | 08.03.2017 | Debris | Kudlov | Zlín | II |
| 70 | 08.03.2017 | Debris | Želechovice - Paseky | Zlín | II |
| 71 | 08.03.2017 | Debris | Újezd u Val. Klobouk | Vsetín | II |
| 72 | 08.03.2017 | Debris | Míkovice nad Olšavou | Uherské Hradiště | II |
| 73 | 13.03.2017 | Debris | Luhačovice | Zlín | II |
| 74 | 13.03.2017 | Debris | Mírov | Šumperk | II |
| 75 | 13.12.2016 | Debris | Zlaté Hory | Jeseník | I |
| 76 | 13.03.2017 | Debris | Fryšták | Zlín | II |
| 77 | 21.02.2017 | Debris | Luhačovice | Zlín | II |
| 78 | 16.02.2017 | Debris | Zbraslavice | Kutná Hora | II |
| 79 | 25.01.2017 | Debris | Krásný Les u Frýdlantu | Liberec | II |
| 80 | 14.03.2017 | Brood sample | Červenka | Olomouc | II |
| 81 | 27.03.2017 | Brood sample | Vnorovy | Uherské Hradiště | II |
| 83 | 03.04.2017 | Brood sample | Starý Hrozenkov | Uherské Hradiště | II |
| 84 | 28.04.2017 | Brood sample | Hynčina | Šumperk | II |
| 85 | 28.04.2017 | Brood sample | Vranová Lhota | Svitavy | II |
| 86 | 02.05.2017 | Brood sample | Semetín | Vsetín | II |
| 87 | 02.05.2017 | Brood sample | Burešov | Zlín | II |
| 88 | 13.03.2017 | Debris | Ostrava Plesná | Ostrava - město | II |
| 89 | 13.03.2017 | Debris | Podolí - Mohelnice | Šumperk | II |
| 90 | 14.03.2017 | Debris | Lukov | Zlín | II |
| 91 | 14.03.2017 | Debris | Kašava | Zlín | II |
| 92 | 14.02.2017 | Debris | Borotice | Příbram | I |

| | | | | | |
|---|---|---|---|---|---|
| 93 | 13.03.2017 | Debris | Pozlovice | Zlín | II |
| 94 | 13.03.2017 | Debris | Poteč | Zlín | II |
| 95 | 17.03.2017 | Debris | Dolní Ves | Zlín | II |
| 96 | 21.03.2017 | Debris | Dolní lhota | Ostrava - město | II |
| 97 | 21.03.2017 | Debris | Kelníky | Zlín | II |
| 98 | 29.03.2017 | Brood sample | Vizovice | Zlín | I |
| 99 | 13.03.2017 | Debris | Město Libavá | Olomouc | II |
| 100 | 21.03.2017 | Debris | Trnava u Zlína | Zlín | II |
| 101 | 20.03.2017 | Debris | Hořany | Most | II |
| 102 | 24.04.2017 | Debris | Partutovice | Přerov | II |

Supplementary Figures S1: Original PCR gels for individual samples and ERIC I and ERIC II reference strains. The numbers above lines represent the IDs of individual samples. Symbol X means error in sample loading.

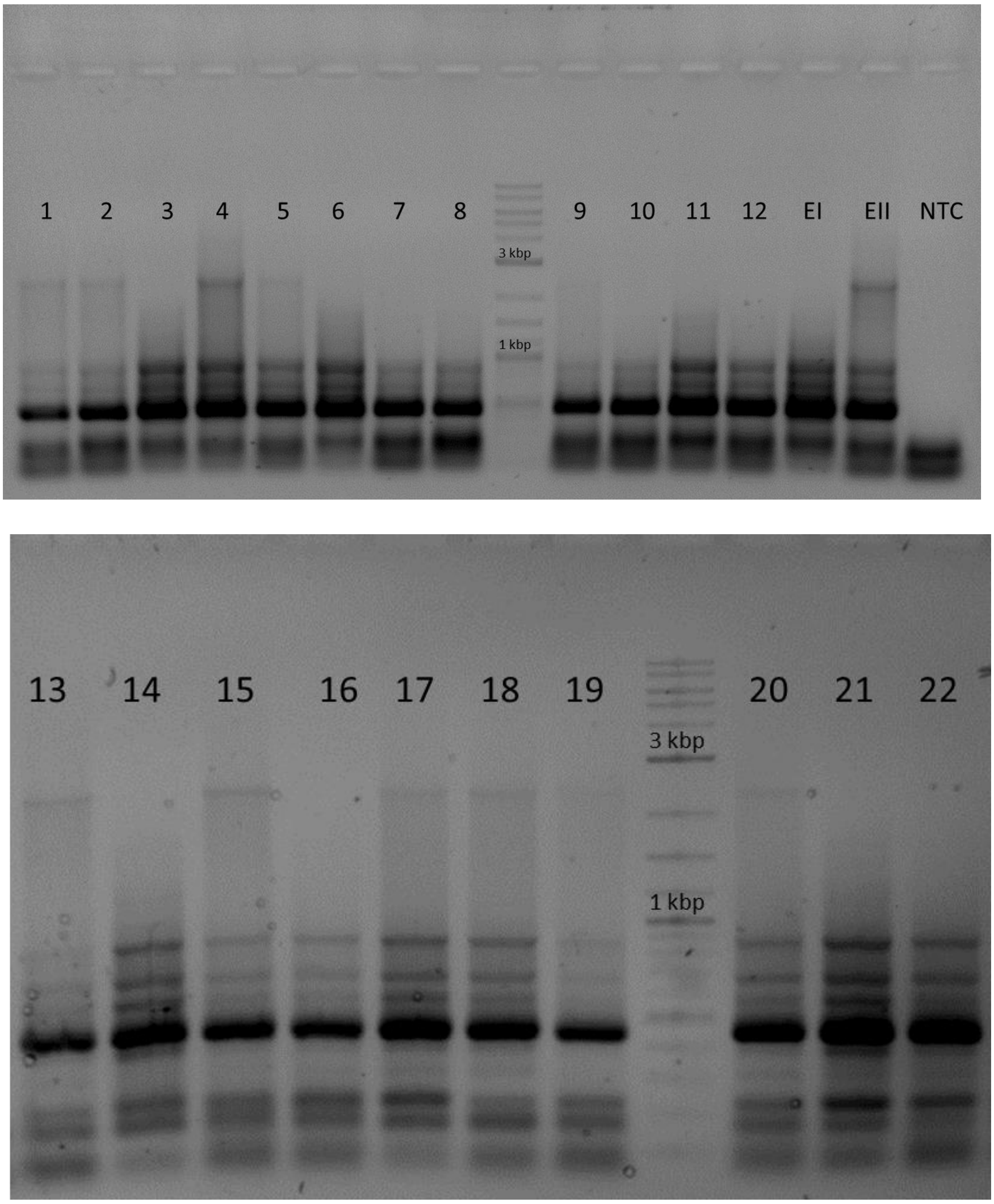

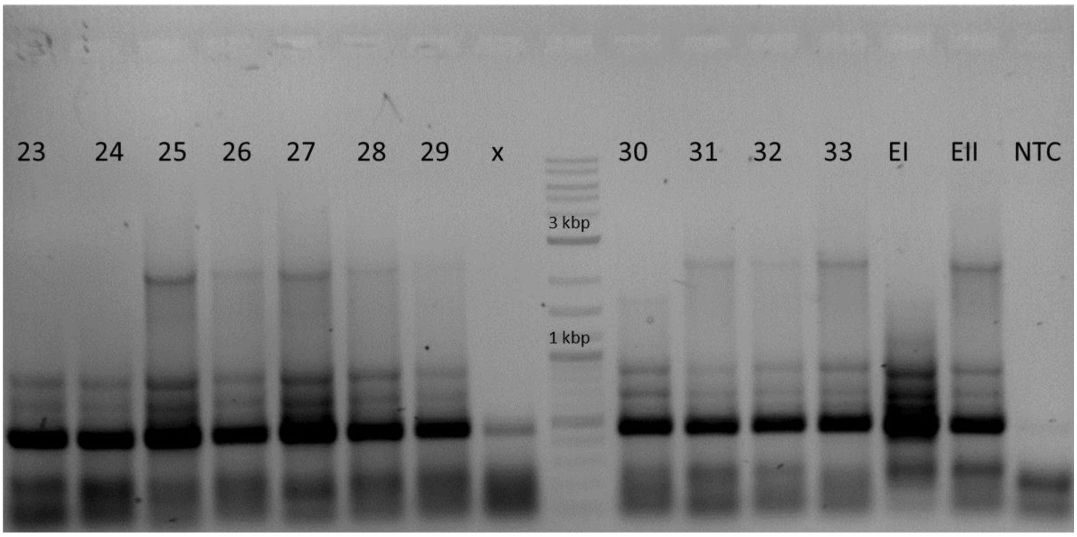
23
24
25
26
27
28
29
x
3 kbp
1 kbp
30
31
32
33
EI
EII
NTC

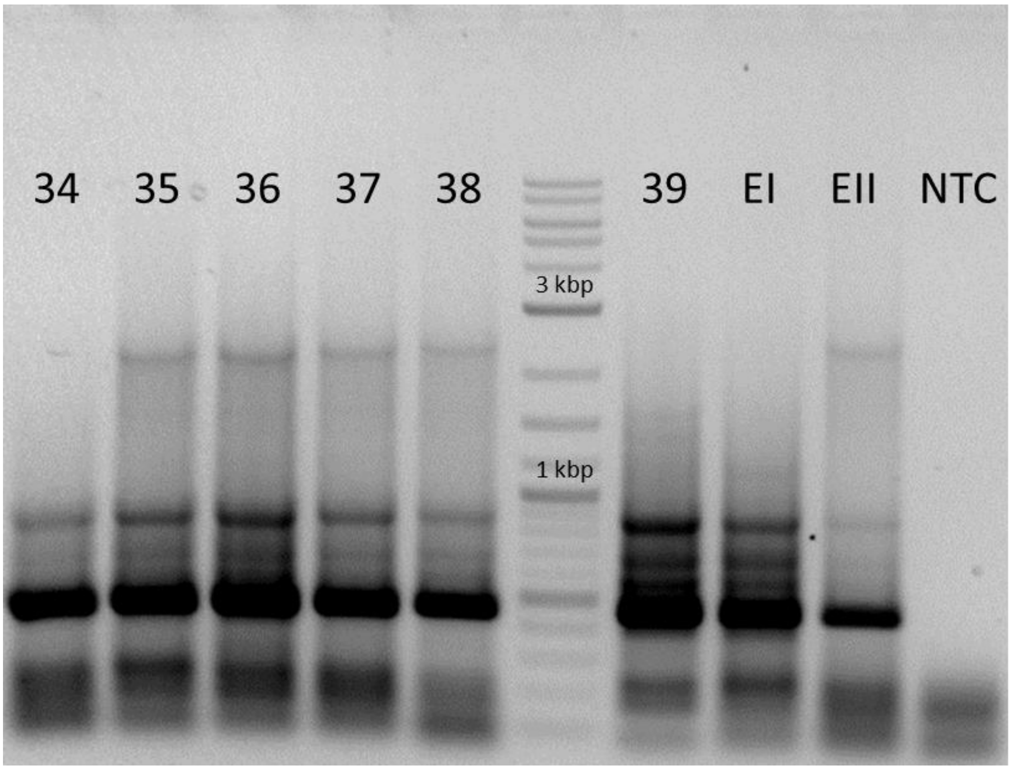
34
35
36
37
38
3 kbp
1 kbp
39
EI
EII
NTC

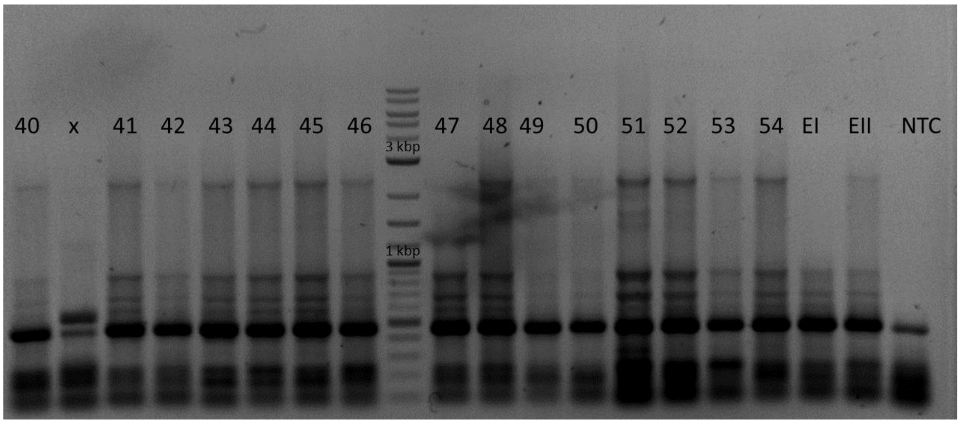
40
x
41
42
43
44
45
46
3 kbp
1 kbp
47
48
49
50
51
52
53
54
EI
EII
NTC

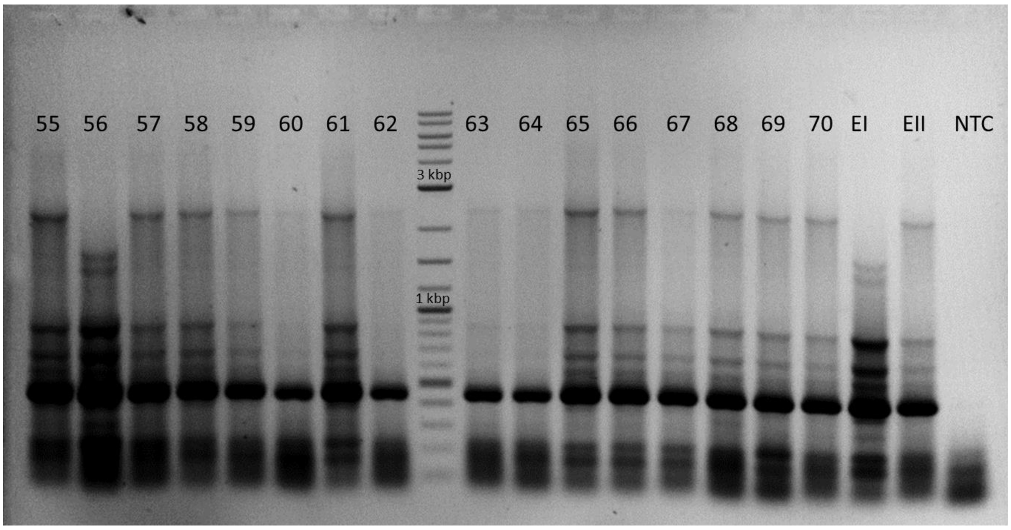
55 56 57 58 59 60 61 62 63 64 65 66 67 68 69 70 EI EII NTC
3 kbp
1 kbp

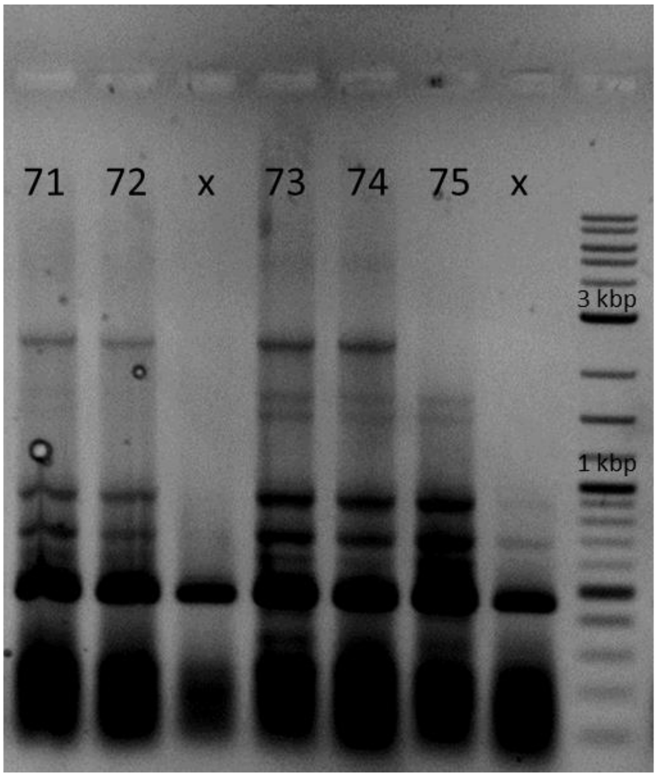
71 72 x 73 74 75 x
3 kbp
1 kbp

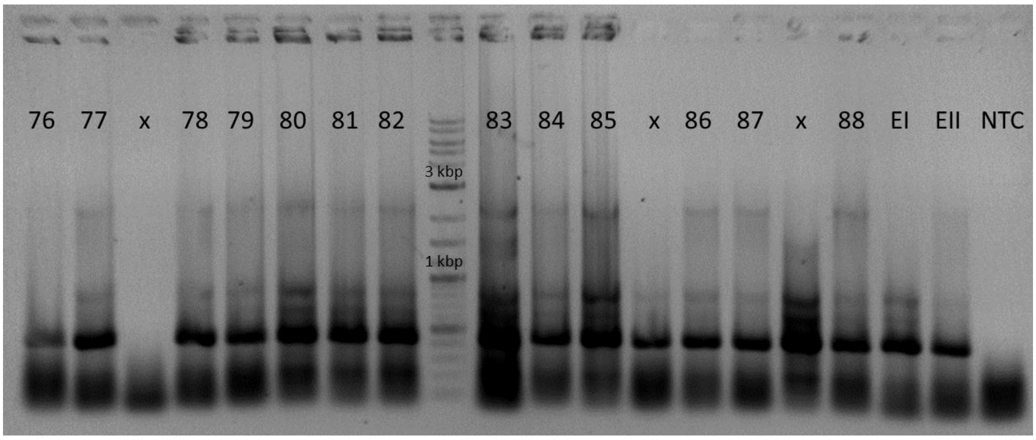
76 77 x 78 79 80 81 82 83 84 85 x 86 87 x 88 EI EII NTC
3 kbp
1 kbp

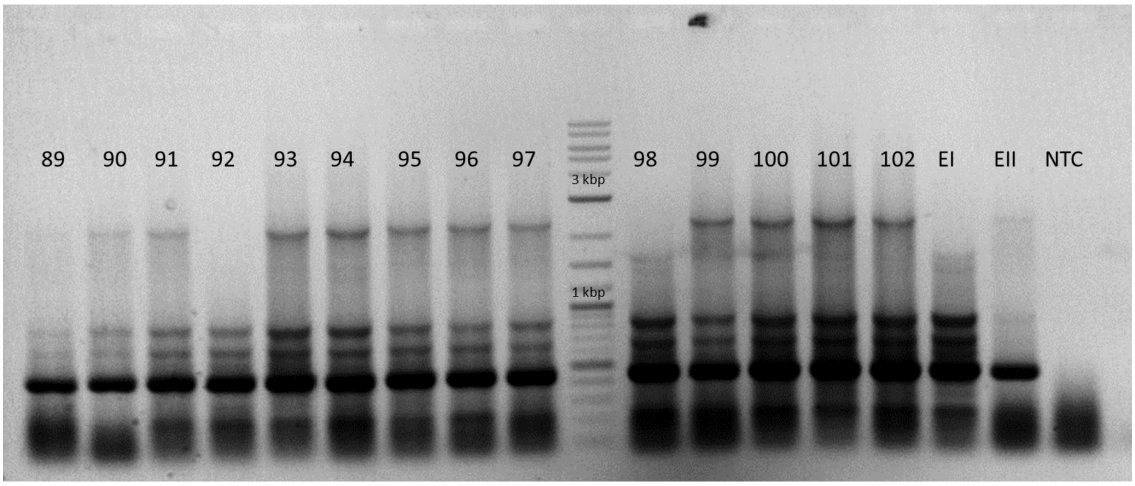
89
90
91
92
93
94
95
96
97
3 kbp
1 kbp
98
99
100
101
102
EI
EII
NTC
